# Supplementary material for: The Global Financial Crisis and Overweight among Children of Single Parents: A Nationwide 10-Year Birth Cohort Study in Japan
Source: Int J Environ Res Public Health. 2019 Mar 20;16(6):1001. doi: 10.3390/ijerph16061001 (PMC6466597; doi:10.3390/ijerph16061001)
Supplement: Supplementary file 1 [file ijerph-16-01001-s001.pdf]

Table S1. Odds ratio (OR) and 95% confidence interval (CI) for risk of overweight relative to normal weight by gender (Full results with estimated odds ratios and confidence intervals for the adjusted covariates)

	Model 1				Model 2			
	Boys		Girls		Boys		Girls	
	OR	(95% CI)	OR	(95% CI)	OR	(95% CI)	OR	(95% CI)
**Age (years)**	1.02	(1.01-1.04)	0.99	(0.97-1.00)	1.02	(1.01-1.04)	0.99	(0.97-1.00)
**Parenthood**								
Two parents	1.00 (Ref.)		1.00 (Ref.)		1.00 (Ref.)		1.00 (Ref.)	
Single parent	1.14	(0.97-1.33)	1.09	(0.93-1.28)	0.97	(0.81-1.15)	0.94	(0.79-1.12)
**Step term**								
Before September 2008	1.00 (Ref.)		1.00 (Ref.)		1.00 (Ref.)		1.00 (Ref.)	
After September 2008	1.62	(1.51-1.72)	1.26	(1.18-1.35)	1.62	(1.51-1.73)	1.26	(1.18-1.35)
**Interaction between single parent in 2008 and step term**								
Two parents *September 2008	1.00 (Ref.)		1.00 (Ref.)		1.00 (Ref.)		1.00 (Ref.)	
Single parents *September 2008	1.10	(0.92-1.30)	1.23	(1.04-1.45)	1.10	(0.93-1.31)	1.23	(1.04-1.46)
**Household income quartile before 2008**								
1 (lowest)					0.99	(0.87-1.12)	1.06	(0.93-1.20)
2					1.05	(0.93-1.18)	0.99	(0.87-1.12)
3					0.93	(0.83-1.05)	0.92	(0.81-1.04)
4 (highest)					1.00 (Ref.)		1.00 (Ref.)	
**>30% negative income change during economic crisis**								
No					1.00 (Ref.)		1.00 (Ref.)	
Yes					1.10	(0.96-1.25)	1.02	(0.88-1.17)
**Mother's education**								
Junior high shool					1.00 (Ref.)		1.00 (Ref.)	
High School					0.70	(0.57-0.86)	0.62	(0.50-0.77)
Vocational School					0.64	(0.52-0.79)	0.50	(0.41-0.63)
Higher Education					0.62	(0.49-0.78)	0.46	(0.36-0.58)
**Father's education**								
Junior high shool					1.00 (Ref.)		1.00 (Ref.)	
High School					0.97	(0.82-1.14)	0.91	(0.78-1.10)
Vocational School					0.92	(0.77-1.10)	0.87	(0.75-1.08)
Higher Education					0.75	(0.63-0.90)	0.76	(0.65-0.94)
**Mother's age**								
< 20 years					1.00 (Ref.)		1.00 (Ref.)	
21-25 years					0.84	(0.54-1.30)	1.10	(0.63-2.25)
26-30 years					0.88	(0.57-1.37)	1.18	(0.62-2.24)

>30 years	0.97	(0.63-1.52)	1.24	(0.65-2.35)
**Father's age**				
< 20 years	1.00 (Ref.)		1.00 (Ref.)	
21-25 years	2.25	(1.17-4.33)	0.79	(0.36-1.77)
26-30 years	1.90	(0.99-3.68)	0.87	(0.39-1.95)
>30 years	2.21	(1.14-4.27)	1.06	(0.47-2.38)
**Residential area**				
20 designated cities	1.00 (Ref.)		1.00 (Ref.)	
other cities	1.07	(0.98-1.18)	1.16	(1.05-1.27)
Rural	1.14	(0.99-1.32)	1.41	(1.22-1.64)
**Three generation household**				
No	1.00 (Ref.)		1.00 (Ref.)	
Yes	1.39	(1.27-1.52)	1.27	(1.16-1.39)

A generalized estimating equation model with an exchangeable correlation structure was used for the analysis
Robust standard errors were used to calculate 95% confidence intervals
Model 2 was adjusted for household income quartile before 2008, onset of 30 % or more negative income change during economic crisis, mother's education, father's education, mother's age at birth, father's age at birth, residential area, and three generation household

Table S2. Odds ratio (OR) and 95% confidence interval (CI) for risk of overweight relative to normal weight by gender and parental reception of social support

	Boys				Girls			
	Having social support		No support		Having social support		No support	
	OR	(95% CI)	OR	(95% CI)	OR	(95% CI)	OR	(95% CI)
**Changes in single parental status between 2008 and 2009**								
Two parents	1.00 (Ref.)		1.00 (Ref.)		1.00 (Ref.)		1.00 (Ref.)	
Single parent	0.93	(0.75-1.16)	1.00	(0.75-1.33)	0.94	(0.75-1.18)	0.91	(0.69-1.20)
**Step term**								
Before September 2008	1.00 (Ref.)		1.00 (Ref.)		1.00 (Ref.)		1.00 (Ref.)	
After September 2008	1.59	(1.45-1.74)	1.66	(1.50-1.82)	1.27	(1.15-1.39)	1.26	(1.14-1.38)
**Interaction between single parent in 2008 and step term**								
Two parents *September 2008	1.00 (Ref.)		1.00 (Ref.)		1.00 (Ref.)		1.00 (Ref.)	
Single parent*September 2008	1.01	(0.80-1.28)	1.25	(0.96-1.63)	1.18	(0.94-1.48)	1.31	(1.00-1.71)

A generalized estimating equation model with an exchangeable correlation structure was used for the analysis

Robust standard errors were used to calculate 95% confidence intervals

Model 2 was adjusted for household income quartile before 2008, onset of 30 % or more negative income change during economic crisis, mother's education, father's education, mother's age at birth, father's age at birth, residential area, and three generation household
